# Genetic and Ecotypic Differentiation in a Californian Plant Polyploid Complex (*Grindelia*, Asteraceae)

**DOI:** 10.1371/journal.pone.0095656

**Published:** 2014-04-22

**Authors:** Abigail J. Moore, William L. Moore, Bruce G. Baldwin

**Affiliations:** 1 Institut für Spezielle Botanik und Botanischer Garten, Johannes Gutenberg-Universität Mainz, Mainz, Germany; 2 Department of Integrative Biology and Jepson Herbarium, University of California, Berkeley, California, United States of America; 3 David Eccles School of Business, University of Utah, Salt Lake City, Utah, United States of America; The University of Queensland, St. Lucia, Australia

## Abstract

Studies of ecotypic differentiation in the California Floristic Province have contributed greatly to plant evolutionary biology since the pioneering work of Clausen, Keck, and Hiesey. The extent of gene flow and genetic differentiation across interfertile ecotypes that span major habitats in the California Floristic Province is understudied, however, and is important for understanding the prospects for local adaptation to evolve or persist in the face of potential gene flow across populations in different ecological settings. We used microsatellite data to examine local differentiation in one of these lineages, the Pacific Coast polyploid complex of the plant genus *Grindelia* (Asteraceae). We examined 439 individuals in 10 different populations. The plants grouped broadly into a coastal and an inland set of populations. The coastal group contained plants from salt marshes and coastal bluffs, as well as a population growing in a serpentine grassland close to the coast, while the inland group contained grassland plants. No evidence for hybridization was found at the single location where adjacent populations of the two groups were sampled. In addition to differentiation along ecotypic lines, there was also a strong signal of local differentiation, with the plants grouping strongly by population. The strength of local differentiation is consistent with the extensive morphological variation observed across populations and the history of taxonomic confusion in the group. The Pacific Clade of *Grindelia* and other young Californian plant groups warrant additional analysis of evolutionary divergence along the steep coast-to-inland climatic gradient, which has been associated with local adaptation and ecotype formation since the classic studies of Clausen, Keck, and Hiesey.

## Introduction

Ecotypes or ecological races have been widely studied in order to understand the processes of adaptation and diversification [1], [2], [3], [4], [5], [6]. Different ecotypes are assumed to possess different morphological or physiological adaptations to their respective habitats. Ecotypes, whether they are considered to belong to a single species or to a complex of closely related species (or whether both taxonomic possibilities have been explored), are often still in contact with each other, are able to exchange genes, and may still be subject to many of the same selection pressures that led to their divergence. Thus, whether or not the ecotypes in question will eventually become fully distinct species, studying them gives us the opportunity to observe the processes leading to diversification, which are ordinarily obscured by subsequent divergence and dispersal in well diagnosed species [7], [8], [9].

Ecotypes have been widely investigated in both plants and animals, with some particularly well-studied examples in animals including *Timema cristinae* Vickery walking sticks associated with different chaparral shrubs (*Ceanothus spinosus* Nutt. and *Adenostoma fasciculatum* Hook. & Arn.) [10], [11], [12] and many different groups of sticklebacks, including lake and stream ecotypes in *Gasterosteus aculeatus* L. (three-spined stickleback) [13], oceanic and freshwater ecotypes in *G. aculeatus* [14], and freshwater and marine ecotypes in *Pungitius pungitius* L. (nine-spined stickleback) [15].

One of the best-studied patterns of ecotypic differentiation in plants is that between coastal and inland ecotypes [6]. Turesson [1] first proposed the concept of ecotypes after discovering that coastal and inland plants of the same species retained their distinct morphologies when grown in a common garden. Coastal and inland ecotypes, among others, were also examined in the classic work of Clausen et al. [2], [4]. More recently, coastal ecotypes were found to have originated multiple times independently from more widespread, inland ecotypes in both *Eucalyptus globulus* Labill. [16] and the *Senecio lautus* G.Forst. ex Willd. complex [17]. The evolution of coastal and inland ecotypes of *Mimulus guttatus* DC. has been extensively studied [18], [19], and reproductive isolation between the ecotypes was found [18].

The California Floristic Province (CA-FP) has long been recognized as an area where many groups of plants and animals have undergone recent diversification, in part reflected by the large number of closely related, but often ecologically divergent, species that are endemic to the area [20], [21], [22], [23]. Classic biosystematic studies examined groups of plant ecotypes or closely related species in the CA-FP [2], [3], [4], [24], [25], [26]. In some cases, particularly among annual groups, the ecologically distinct entities are sufficiently divergent to be considered well-diagnosed species (e.g., in *Clarkia* Pursh [26], *Collinsia* Nutt. [27], *Downingia* Torr. [28], and *Layia* Hook. & Arn. ex DC. [29]). In other cases, ecological change has been accompanied primarily by evolutionary changes in vegetative morphology that could be easily mistaken for phenotypic plasticity, even though species-level classifications have also been proposed (e.g., in *Achillea* L. [2], [30] and *Mimulus* L., [18], [19]).

One group that shows considerable ecological and morphological diversity within the CA-FP is the New World genus *Grindelia* Willd. (Asteraceae: Astereae). Throughout its range in North America and South America, members of the genus occupy a wide variety of open, predominantly xeric habitats, such as grasslands, deserts, and early successional sites, as well as coastal bluffs and salt marshes. One clade of *Grindelia* is restricted to far western North America, from Baja California, Mexico, to British Columbia, Canada and has its center of morphological and ecological diversity in the CA-FP. This Pacific Clade was well-supported in molecular analyses of nuclear ribosomal DNA sequences, although resolution within the clade was poor due to lack of sequence divergence [31]. In addition, the clade appears to be distinguished from the remainder of *Grindelia* by a chromosomal rearrangement [32].

Given the lack of sequence divergence among members of the Pacific Clade, the group as a whole appears to be quite young [31]. Despite the recent diversification of the clade, its members occupy almost the entire range of habitats occupied by the rest of the much older genus including coastal bluffs, dry grasslands, salt- and brackish-water marshes, and serpentine barrens. Plants growing in different habitats exhibit morphological and phenological differences. They vary in stature (from 30 to 200 cm tall), woodiness (from not at all woody to having woody branches more than 1 m long), head shape and size, leaf and phyllary morphology, degree of succulence, amount of resin, and flowering time (from early summer to fall). These differences appear to be genetic, as they persist when the plants are grown in a common garden [33], [34]. Although there is clearly much morphological variation within Pacific *Grindelia*, and plants from two different habitats often have striking morphological differences, it is generally very difficult to draw clear boundaries between putative taxa when they are examined across their entire ranges. This problem is reflected in the variety of taxonomic hypotheses that have been proposed for the group, ranging from treatment of the plants as a group of 16 related species [35] to considering almost all members of the Pacific Clade as one variable species that shows much local differentiation [36], with most authors following a middle ground [37], [38], [39], [40].

The phylogenetic difficulties are compounded by variation in ploidy. The Pacific Clade includes both diploids and tetraploids [33]. The diploids tend to be rather small plants (30–60 cm tall) of grasslands and occur mostly towards the edges of the CA-FP or outside of its boundaries. Most of the ecological and morphological variation within the Pacific Clade is among its tetraploid members, which occur along the Pacific Coast and throughout a large part of the CA-FP. The tetraploids have been considered to be autotetraploids based on observations of their chromosome pairing at meiosis [33]. All species are obligate outcrossers [33] and the flower heads are attractive to insect pollinators [41], [42]. In the greenhouse, all crosses at the same ploidy level are successful and progeny do not show a reduction in pollen fertility [33]. Although the tetraploids are interfertile [33], the number of tetraploidization events and the diploid parent(s) of the tetraploids are both unknown.

**Figure 1:**
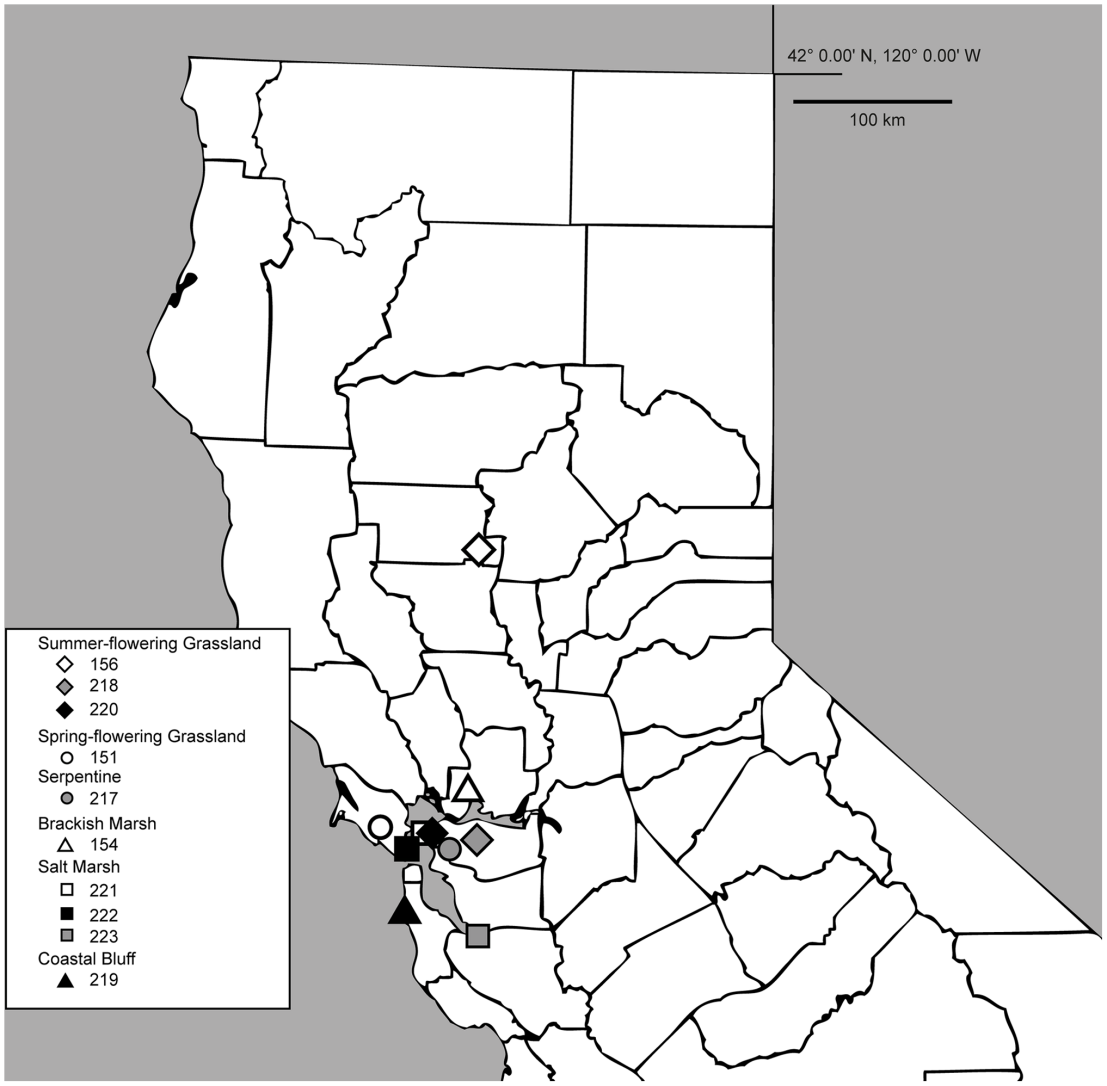
Locations of the ten sampled populations in northern California.

Here we examine gene flow between populations and ecotypic division in Pacific *Grindelia* using microsatellite data. Ten populations, with 29 to 50 individuals per population, were sampled (Table 1; Fig. 1). We chose to sample plants from a wide variety of habitats in a relatively small geographic area instead of sampling plants from fewer habitats throughout a larger area in order to be able to examine local interactions. This study was designed to determine whether the populations constitute larger, genetically cohesive groups and, if so, if these groups are ecologically explicable. In addition, we examined the amount of gene flow that has occurred between populations and whether there are boundaries to gene flow along ecological lines.

**Table 1:** Voucher information for the samples included in the study.

Habitat	Population number	N^1^	Voucher^2^	county^3^	Collection location
serpentine grassland	151	30	*Moore 818*	Marin	Mount Tamalpais State Park
brackish marsh	154	30	*Moore and Park 819*	Solano	Hill Slough Wildlife Area
grassland	156	29	*Moore et al. 822*	Glenn	Sacramento National Wildlife Refuge
grassland	217	50	*Moore et al. 861*	Contra Costa	Wildcat Canyon Regional Park
grassland	218	50	*Moore et al. 862*	Contra Costa	Mount Diablo State Park
coastal bluffs	219	50	*Moore et al. 863*	San Mateo	Montara State Beach
grassland	220	50	*Moore et al. 864*	Contra Costa	Point Pinole Regional Park
salt marsh	221	50	*Moore et al. 865*	Contra Costa	Point Pinole Regional Park
salt marsh	222	50	*Moore et al. 866*	Marin	China Camp State Park
salt marsh	223	50	*Moore and Park 870*	Alameda	Don Edwards San Francisco Bay National Wildlife Refuge

1 N: the number of individuals sampled in the population;

2 voucher specimens are deposited in the Jepson Herbarium (JEPS);

3 all counties are in California.

## Materials and Methods

### Ethics Statement

All sampled populations of Pacific *Grindelia* were located on protected land, but none is protected under endangered species legislation. Permits were acquired from the following authorities for sampling on the land that they manage: the California Department of Fish and Game (Hill Slough Wildlife Area, population 154), California State Parks (China Camp State Park, population 222; Montara State Beach, population 219; Mount Diablo State Park, population 218; Mount Tamalpais State Park, population 151), the East Bay Regional Park District (Point Pinole Regional Park, populations 220 and 221; Wildcat Canyon Regional Park, population 217), the Sacramento National Wildlife Refuge Complex (Sacramento National Wildlife Refuge, population 156), and the San Francisco Bay National Wildlife Refuge Complex (Don Edwards San Francisco Bay National Wildlife Refuge, population 223). All necessary permits were obtained for the described study, which complied with all relevant regulations.

### Sampling

Between 29 and 50 individuals from each of ten populations were sampled, for a total of 439 individuals (Table 1). All populations were tetraploid with the exception of one diploid population, 218 from Mount Diablo, growing in grassland The ploidy of a given population is easily determined with microsatellites, as diploids have one or two alleles at each locus, while tetraploids have up to four alleles per locus.

The term population is used to refer to a group of plants growing in the same habitat in a given area. In non-wetland habitats, the boundaries of the populations were quite distinct, and samples were obtained from throughout the entire area where the plants occurred at a collection locality. In wetland habitats, the plants occurred in much more extensive stands, and we were only able to sample plants from part of the area occupied by *Grindelia* at a given site. All collection localities were separated from one another by large areas of unsuitable habitat, except for the two localities at Point Pinole (populations 220 and 221), where morphologically different populations were found within ca. 100 m of each other in grassland and saltmarsh habitats. This distance is likely within the flight distance of their bee pollinators [43]. No morphological intermediates were observed at this location.

### Microsatellite Amplification and Scoring

Sequences of microsatellite-containing loci were obtained using the protocol of [44] with DNA from the specimen *Moore, Silviera, and Anderson 551* (JEPS), collected in the Sacramento National Wildlife Refuge, near the site of collection of population 156. Primer sequences for the variable loci are described in [45]. In this study, we used the six primer pairs GRIN024, GRIN026, GRIN035, GRIN045, GRIN068, and GRIN113. The remaining five primer pairs described in [45] had results that were difficult to interpret, due to the length of the PCR product or the number of bands produced.

DNA was extracted from fresh or silica-dried material using the Qiagen Plant Mini Kit (Qiagen, Inc., Valencia, California). Samples were ground directly in AP1 extraction buffer using a mortar and pestle or ground dry using glass beads in a Mini-Bead-Beater-16 (BioSpec Products, Inc., Bartlesville, Oklahoma).

Most loci were amplified with component-based PCR in 25 µl reactions with 1× ThermoPol reaction buffer (New England Biolabs, Inc., Ipswich, Massachusetts), 1.5 units of *Taq* polymerase (New England Biolabs), 0.4 µM each primer, 0.6 mM dNTPs, 0.5 µg BSA, and 3 µl DNA that was diluted 1∶10 from the concentration of the originally extracted DNA. Loci 045 and 113 were amplified using AccuPower PCR PreMix (Bioneer Inc., Alameda, California) in 20 µl reactions using 0.375 µM concentration of each primer and 3 µl of DNA at 1∶10 dilution. The touchdown PCR program of [46] was used, with annealing temperatures of 55–45°C. In all cases, the forward primer was fluorescently labeled (HEX for loci 024, 045, and 113 and 6FAM for loci 026, 035, and 068).

Samples were run on ABI 3730×l capillary sequencing machines (Applied Biosystems, Inc., Foster City, CA, U.S.A.) at the U.C. Berkeley DNA Sequencing Facility using the GeneScan 500 ROX size standard (Applied Biosystems). Samples were scored using Peak Scanner v.1.0 software (Applied Biosystems). A subset of individuals (ca. 5%) was run twice to ensure that amplification and scoring of alleles were consistent across runs.

All individuals produced between 1 and 4 score-able bands at each locus (or between 1 and 2 score-able bands in the diploid population 218), with the exception of individuals 9 and 23 from population 223 (salt marsh), for which locus 024 did not amplify; individuals of 4, 7, and 14 from population 222 (also salt marsh), which produced 5 bands at locus 026; and individual 10 from the diploid population 218 (grassland), which produced 4 bands at locus 024.

Microsatellite data were scored by recording the presence or absence of the alleles (phenotypic scoring), instead of by attempting to determine how many copies of each allele were present in a given individual (genotypic scoring). We chose to perform phenotypic instead of genotypic scoring because at least some individuals at each of the different loci had stutter bands or had peaks that were overloaded, so the full area of the peaks could not be accurately determined. The scored microsatellite data have been deposited in Dryad (doi:10.5061/dryad.rv524).

### Data Analysis

Dice’s similarity coefficient [47] was used to create a matrix of distances between each pair of individuals. As Dice’s similarity coefficient does not differentiate between loci (and thus allows an arbitrarily large number of alleles per locus), all alleles were included for the individuals with five alleles at locus 026. Diploid and tetraploid populations were treated identically. Principal Coordinates Analysis (PCO) was performed on these distances using R [48] in order to create the best low dimensional visual representation of these data as well as to provide a set of uncorrelated independent variables for discriminant analysis and hierarchical analysis of molecular variance.

Discriminant analyses were performed using SPSS version 20.0.0.1 (IBM Corp., Armonk, New York) to determine whether populations and groups differed significantly in terms of the principal coordinates that had corresponding eigenvalues greater than zero. Discriminant analyses were performed with the plants grouped two different ways: into populations (ten groups) and into the two groups from the *structure* [49] analysis with *K* = 2, with the two intermediate populations not included. After the discriminant analysis in which the plants from seven of the ten populations were grouped according to the *structure* groups, the discriminant classification function was used to classify the plants from the remaining three populations into one of the existing groups. A finding that both discriminant analyses are significant would indicate not only that the populations are significantly different, but also that there is a hierarchical structure to these differences, where the larger groups are also significantly different from each other.

SPAGeDi v. 1.3a [50] was used to calculate ρ [51], an analog of *F*_ST_ that is calculated using allelic phenotypes. ρ is independent of both the amount of double reduction and the degree of inbreeding. It can therefore be used to compare diploids and tetraploids [51]. ρ was calculated for each locus separately and for all loci combined across all individuals. Diploids and tetraploids were treated identically. A PCO was performed using the ρ values for all loci combined as a distance measure. The plots of the population centroids created from the PCO analysis of Dice’s similarity coefficients, the discriminant function space, and the PCO analysis of ρ were compared by calculating the correlation of the distances between all pairs of points using R.

*Structure* [49] was used to examine the division of individuals into groups. Two different types of analyses were performed. First, analyses were performed assuming the genotypes were known unambiguously in order to determine the optimal number of groups into which the individuals should be divided. Second, once the optimal number of groups was determined, analyses were conducted that took genotypic uncertainty into account, for the optimal number of populations found in the first set of analyses. It was necessary to run two sets of analyses because *Structure* cannot calculate the likelihood values of the individual runs accurately when there is genotypic uncertainty [52], and the likelihood values are needed to choose the optimum number of groups.

The first set of analyses, in which it was assumed that the genotypes were known unambiguously, was performed with data sets in which 4-allele genotypes were created by replacing the unknown alleles with one of the known alleles with equal probability. For example, if an individual had three different alleles at a given locus, the fourth allele had a one-third probability of being a repeat of any of the other three. Diploids were coded with two or four copies of each allele, depending on whether they were heterozygous or homozygous at a given locus. Four different data sets, with different random resolutions of the tetraploid genotypes, were constructed. For these analyses, it was assumed that there were no recessive alleles and no ambiguity (RECESSIVEALLELES = 0). The number of groups (*K*) was allowed to vary from 2–15; 20 replicate runs were performed for each value of *K*. Each replicate was run for 100,000 generations preceded by a burn-in period of 30,000 generations. Admixture was allowed, and allele frequencies were independent in the different populations. As the analysis sometimes had trouble converging at higher values of *K*, the two populations with the lowest log likelihood values were removed prior to comparing the likelihood values at the different values of *K*. We also considered the delta K method of Evanno et al. [53], but those results were inconsistent across the four data sets, likely due to the different random resolutions of the genotypes. We chose the number of groups based on the maximum likelihood value.

The second set of analyses, in which genotypic uncertainty was taken into account, was performed with one of the data sets from the first set of analyses. Replicate data sets were not necessary, as individuals with different numbers of the same alleles would be treated identically [54]. The recessive alleles were considered to be present (RECESSIVEALLELES = 1) and the ambiguous allele code (the allele that would normally be recessive) was set to −9, the value for a missing allele, for each of the six loci as recommended by Pritchard et al. [52]. For this analysis, the number of groups (*K*) was set to 2 or to 10, which were the optimal numbers from the previous analysis. Each replicate was run for 100,000 generations preceded by a burn-in period of 10,000 generations. Admixture was allowed, and allele frequencies were independent in the different populations.

For the analyses with SPAGeDi and *structure*, the two individuals lacking locus 024 were coded as having missing data at that locus (ca. 0.08% missing data), while only the four shortest alleles were used for the three individuals with five alleles at locus 026. Each of the five alleles present in those three individuals was also found in other plants, so it was not clear which allele was the extra one and the choice of not using the longest allele was arbitrary.

A hierarchical analysis of molecular variance (AMOVA, [55]) was performed on the squared distances between all pairs of observations (based on the principal coordinates that had corresponding eigenvalues greater than zero) using R. A null distribution was obtained by allocating individuals to a randomly chosen population, holding population sizes constant. Two analyses were conducted; one with the plants grouped according to population and the other with the plants grouped according to the results of the *structure* analysis with *K* = 2 (with only the seven populations that could be unambiguously classified included).

## Results

Across the six loci, 134 alleles in total were found, with the number of alleles per locus ranging from 7 to 31 overall and 3 to 19 within a population (Table 2). For each locus, there was at least one individual that had a single allele, while for five of the six loci there was at least one individual that had four different alleles. The exception was locus 068, likely due to the small number of alleles at that locus (some individuals from other locations were found that had four alleles; A. J. Moore, unpublished data).

**Table 2:** Summary statistics for each locus.

Locus	size range	*N* 1(per pop.)	mean ρ (range)
G024	200–276	21 (5–13)	0.220 (−0.010–0.423)
G026	211–304	31 (10–19)	0.128 (0.027–0.231)
G035	187–235	17 (5–11)	0.260 (0.027–0.588)
G045	387–431	30 (9–17)	0.271 (0.046–0.497)
G068	350–378	7 (3–4)	0.168 (−0.026–0.473)
G113	461–535	28 (8–15)	0.116 (0.008–0.246)

1 *N*: number of alleles.

**Figure 2:**
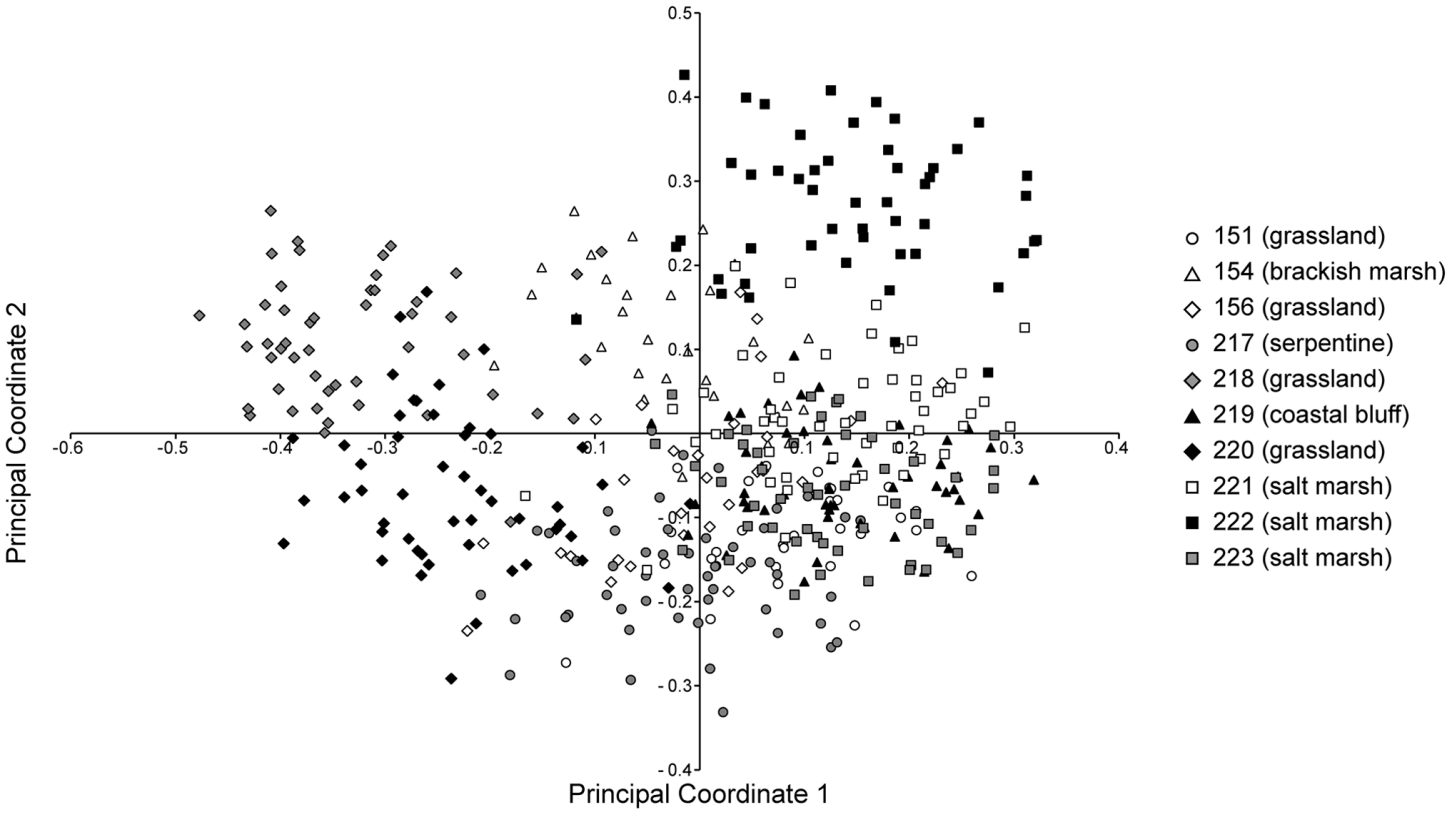
Plot of the first two principal coordinates from the analysis of all 439 individuals. Distances were calculated using Dice’s similarity index. Symbols follow Figure 1.

**Figure 3:**
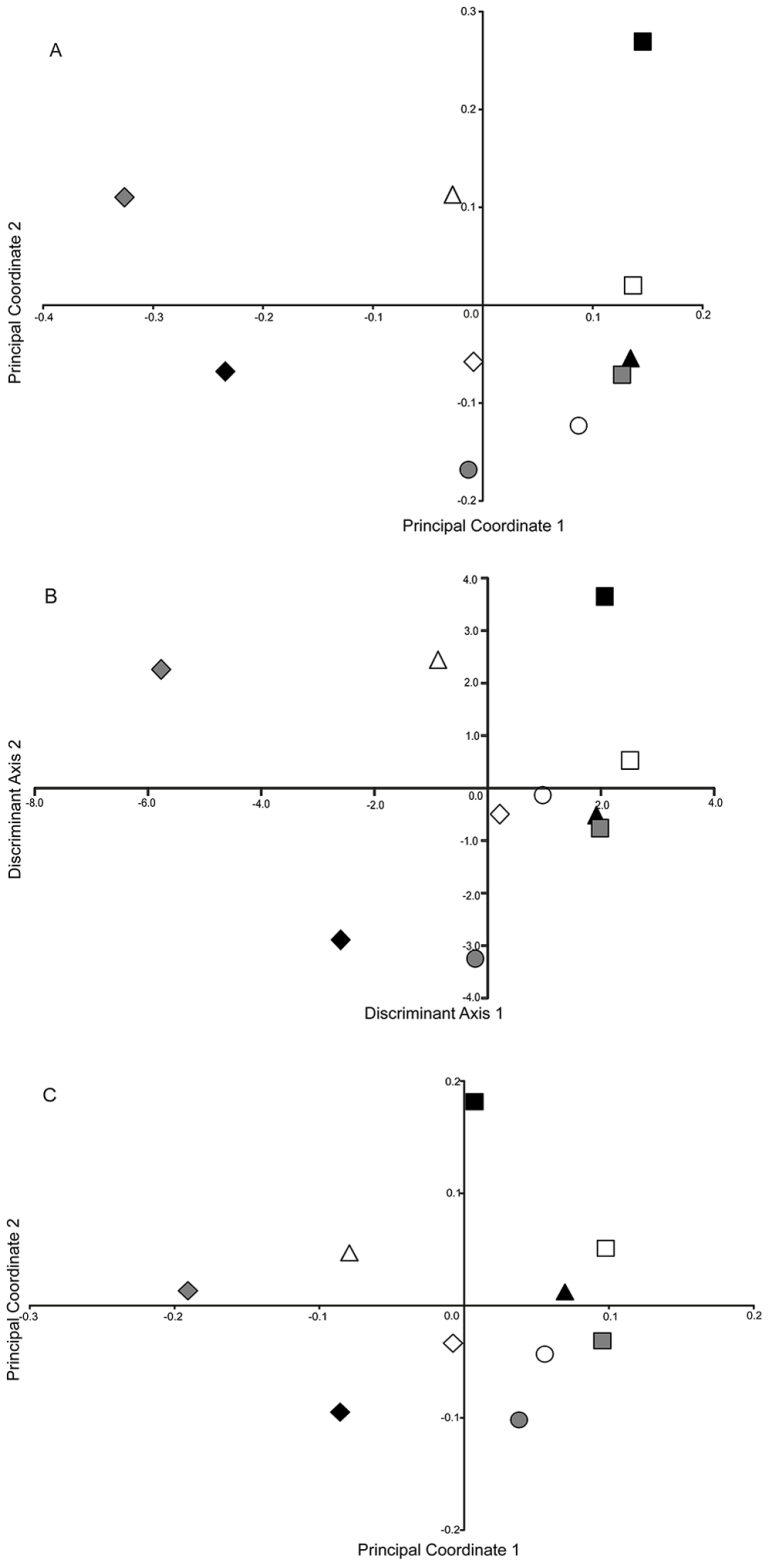
Plots of the populations under three separate analyses. The percent of the variance explained by the first two axes is shown for each analysis. (A) Population centroids from the Principal Coordinates Analysis of the analysis of all individuals using Dice’s similarity index; 15.7% of the variance explained. (B) Population centroids from the discriminant analysis of the Principal Coordinates data; 46.4% of the variance explained. (C) Principal Coordinates Analysis of the ρ data calculated for each population; 66% of the variance explained. Symbols follow Figure 1.

The first two principal coordinates explained 9.69% and 6.04% of the variance in the data, respectively. While these two dimensions explained less than 20% of the variance in the data, they provide the best two-dimensional representation and are shown to help visualize the data. In the plot of those two coordinates (Fig. 2, all individuals; Fig. 3A, population centroids), individuals from the same population grouped together, although there was overlap between populations. The first axis primarily reflected a separation of populations occurring near the coast from those occurring further inland. The populations collected in the salt marsh (221, 222, and 223), on the coastal bluff (219), and in the serpentine grassland (151) had positive values; two of the four (non-serpentine) grassland populations (218, 220) had negative values; and the brackish marsh population (154) and two remaining grassland populations (156 and 217) had intermediate values that partially overlapped with the other groups.

There were 88 principal coordinates that had corresponding eigenvalues greater than zero. In the discriminant analysis, a significant difference among the ten populations based on these 88 principal coordinates was found (χ^2^ test, *p*<0.001); in addition, 98.6% of individuals (all but six) were classified in the correct population (Table 3). The misclassified individuals were fairly evenly distributed across populations, with the exception of population 221 (salt marsh), of which three of the 50 individuals were misclassified as members of population 219 (coastal bluffs). The first two discriminant functions together explained 46.4% of the variance. As expected, the relative positions of the population centroids (Fig. 3B) were quite similar to their positions in the plot of the first two principal coordinates, even though the latter explained only 15.7% of the variance (interpoint distance correlation of 0.89).

**Table 3:** The percentage of individuals from the various populations (rows) that were classified as members of each population (columns) in the discriminant analysis of the principal coordinate data.

	151	154	156	217	218	219	220	221	222	223
151 (serpentine grassland)	100.0	0.0	0.0	0.0	0.0	0.0	0.0	0.0	0.0	0.0
154 (brackish marsh)	0.0	96.7	0.0	0.0	0.0	3.3	0.0	0.0	0.0	0.0
156 (grassland)	0.0	0.0	96.6	0.0	0.0	3.4	0.0	0.0	0.0	6.9
217 (grassland)	0.0	0.0	0.0	100.0	0.0	0.0	0.0	0.0	0.0	0.0
218 (grassland)	0.0	0.0	0.0	0.0	100.0	0.0	0.0	0.0	0.0	0.0
219 (coastal bluff)	0.0	0.0	0.0	0.0	0.0	100.0	0.0	0.0	0.0	0.0
220 (grassland)	0.0	0.0	0.0	0.0	0.0	0.0	100.0	0.0	0.0	0.0
221 (salt marsh)	0.0	0.0	0.0	0.0	0.0	6.0	0.0	92.0	0.0	2.0
222 (salt marsh)	0.0	0.0	0.0	0.0	0.0	0.0	0.0	0.0	100.0	0.0
223 (salt marsh)	0.0	0.0	0.0	0.0	0.0	0.0	0.0	0.0	0.0	100.0

ρ is a measure of population differentiation that is analogous to *F*_ST_ and can be used to compare populations of different ploidy [51]. Its values varied widely across population pairs and loci (Table 2, S1). All loci had some pairs of populations with ρ values less than 0.10 and some pairs of populations with ρ values greater than 0.20. In a PCO analysis of the ρ data, the first two dimensions accounted for 66.0% of the explained variance. The plot of the first two coordinates of this PCO based on ρ (Fig. 3C) was similar to the plot of the population centroids from the PCO analysis based on Dice’s similarity coefficient (interpoint distance correlation of 0.95).

**Figure 4:**
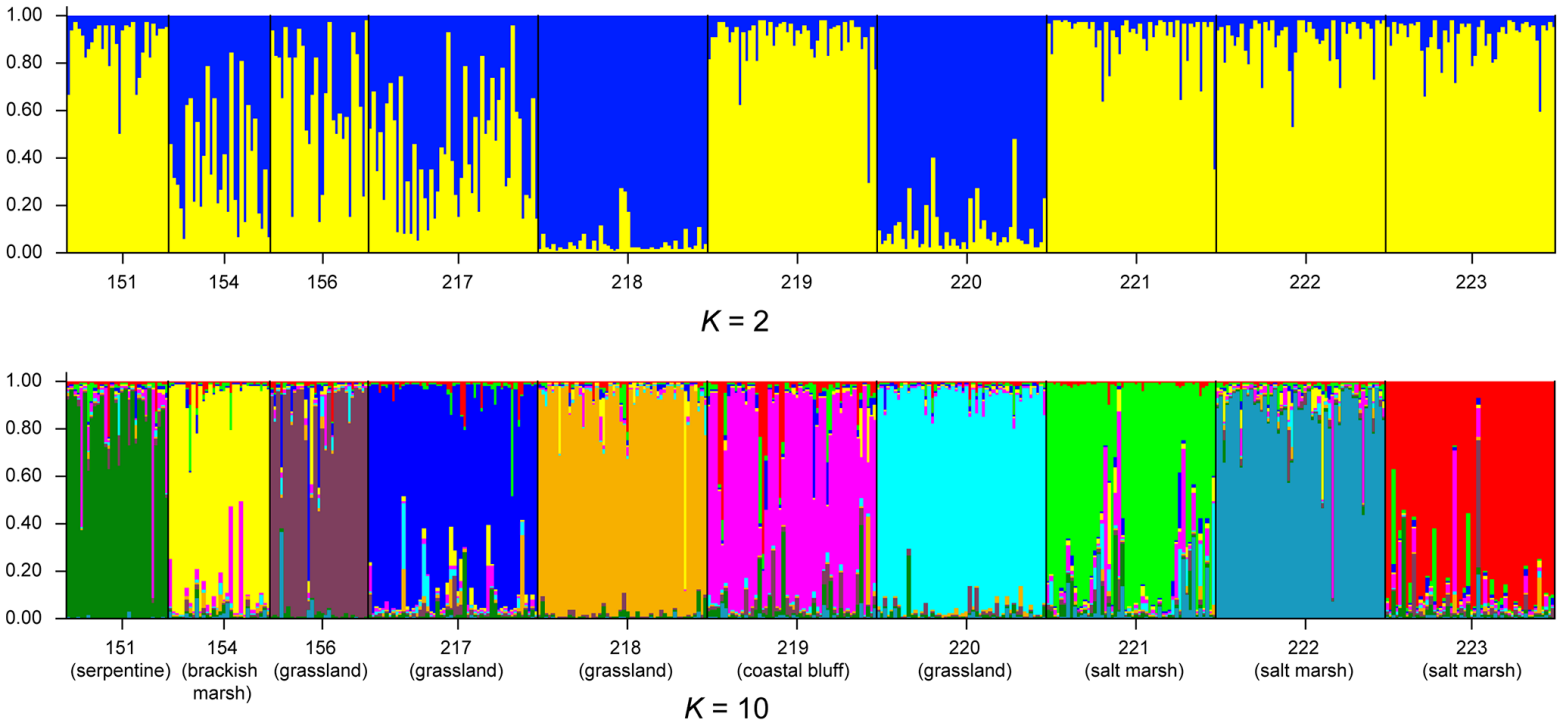
Representative plots from the *structure* analysis, with genotypes assumed to be unambiguous. *K* is the number of groups into which the plants were divided.

In the *Structure* analyses, dividing the plants into ten groups gave the highest likelihood values in all four replicate runs with different randomly created genotypes. The composition of these groups corresponded closely to the sampled populations (Fig. 4). The two sets of *structure* analyses (with and without correcting for allele copy number) gave the same results.

When the number of groups in *structure* was set to two, the distribution of individuals across groups corresponded closely to the division of individuals along the first principal coordinates axis (Fig. 4). Although a division of the plants into two groups had a lower likelihood value than a division of the plants into ten groups, the variance in the likelihood between replicate runs was very low when the number of groups was set to two, indicating that there was also a strong signal in the data at this level. One group (yellow in Fig. 4) contained coastal populations, including serpentine grassland (151), coastal bluffs (219), and salt marsh (221–223) populations. The second group (blue in Fig. 4) contained inland populations with two of the four grassland populations (218 and 220). The three remaining populations, one from a brackish marsh (154) and the remaining two grassland populations (156 and 217) were fairly evenly split between the two groups.

A discriminant analysis was performed on the seven populations that were unambiguously classified in the *structure* analysis when the number of populations was set to two. Grouping the individuals according to the *structure* results explained the data significantly better than leaving them ungrouped (χ^2^ test, *p*<0.0001). In this analysis, 99.4% of individuals (all but two) were correctly classified (Table 4). When the discriminant function was used to classify the individuals in the remaining two populations, the three populations were split between the two groups.

**Table 4:** The percentage of individuals from the two ecotypes and the three intermediate populations (rows) that were classified as members of each ecotype (columns) in the discriminant analysis of the principal coordinate data.

	coastal	inland
coastal (151, 219, 221, 222, 223)	100	0
inland (218, 220)	0.9	99.1
154 (brackish marsh)	83.3	16.7
156 (grassland)	55.2	44.8
217 (grassland)	60.0	40.0

In an AMOVA with all populations included (Table 5), most of the variance (77.1%) was within populations. However, a significant amount of variance was also found among populations (*p* = 0.001). When the plants were divided into coastal and inland populations (according to the *structure* results and the first principal coordinates axis, leaving the three populations out whose classification into these groups was ambiguous), 9.9% of the variance was between coastal and inland groups, 13.5% was among populations within groups, and 76.7% was within populations (*p* = 0.001 for all levels; Table 6).

**Table 5:** AMOVA of microsatellite data, divided according to population, with all ten populations included.

source of variation	d.f.^1^	SS^2^	MS^3^	percentage of variance	Sigma	p^4^
among populations	9	36.71	4.079	22.9	0.082	0.001
within populations	429	123.83	0.29	77.1	0.29	0.001
total	438	160.54				

1 d.f.: degrees of freedom;

2 SS: sum of squares;

3 MS: mean squares;

4 p: significance level.

**Table 6:** AMOVA of microsatellite data, divided according to ecotype and population, with the seven unambiguously classified populations included.

source of variation	d.f.^1^	SS^2^	MS^3^	percentage of variance	Sigma	p^4^
between ecotypes	1	11.9	11.90	9.9	0.023	0.001
among populations within ecotypes	5	16.2	3.24	13.5	0.063	0.001
within populations	323	92.3	0.29	76.7	0.290	0.001
total	330	120.4				

1 d.f.: degrees of freedom;

2 SS: sum of squares;

3 MS: mean squares;

4 p: significance level.

## Discussion

### Local Differentiation

The strongest divisions in the microsatellite data were between populations. These genetic divisions between populations were also supported by the morphological uniformity within populations and morphological differentiation between populations. The populations were structured into coastal and inland groups that are hereafter referred to as ecotypes. At the single site where the two ecotypes came into contact, no gene exchange was observed. Thus, although experimental hybrids between coastal and inland plants are fully fertile [33], barriers to gene flow appear to exist in nature.

Evidence for individual populations being the strongest groups was found in the *structure* analyses, the PCO, which grouped plants from the same population together, and the discriminant analysis, which was able to classify almost all individuals into the correct population. Rho (the analog of *F*_ST_ for polyploids or for cross-ploidal comparisons) was also relatively high between all pairs of populations. The relative genetic isolation of the different populations supports Strother and Wetter’s [36] hypothesis that distinctive patterns of morphological and ecological variation within CA-FP *Grindelia* (“facies”) correspond to local or regional differentiation. Local differentiation was also shown by the morphological differences between populations of *Grindelia* collected in grasslands of California’s Central Valley, which persisted when the plants were grown in a common garden [34].

It is unclear whether this local differentiation in *Grindelia* is due to few opportunities for gene exchange between populations or to selection against migrants (from local adaptation). The idea that lack of opportunity for gene exchange could be a contributing factor is supported by the current isolation of populations of *Grindelia* in this part of its range. Even within areas of apparently suitable upland habitat, *Grindelia* is found in small patches (generally of 20 to 500 individuals) that are generally isolated from other patches by 1 km or more (A. J. Moore, pers. obs.). The major exceptions to this patchy distribution are the marsh plants, which occur in linear populations extending along the banks of sloughs and the shores of San Francisco Bay. Even these large populations are presently separated from each other by areas of unsuitable habitat (although they may have been more connected before the destruction of many of the salt marshes surrounding San Francisco Bay). However, there are also cases (including the two Point Pinole populations included in this study), where two morphologically different populations occur in slightly to entirely different habitats in close proximity to each other. In these cases, the plants are able to maintain their morphological differences (and, in the case of the plants at Point Pinole, their genetic differences), despite the opportunity to interbreed.

### Coastal and Inland Ecotypes

In addition to grouping the plants into populations, there was evidence in the microsatellite data that the populations themselves could be grouped according to distribution and ecology, with a coastal group, an inland group, and three populations that were somewhat intermediate between the two groups. The coastal group consisted of populations growing in the salt marshes surrounding San Francisco Bay (3 populations), coastal bluffs (1), and serpentine grasslands (1). This last population was collected from Mt. Tamalpais, ca. 3 km from the coast, in an area heavily influenced by fog. The inland group consisted of two populations from non-serpentine grasslands. Although we refer to these groups as coastal and inland, increased sampling may indicate that the groups are not divided strictly along coastal and inland lines or that additional coastal or inland groups may be found.

Three populations (154, 156, and 217) were intermediate between the coastal and inland groups in the *structure* analysis and were split between coastal and inland groups in the discriminant analysis. Population 156 was the only population from outside of the San Francisco Bay Area, coming from approximately 175 km north, near the Sacramento River. It grew in vernal pools, areas that are filled with water during the winter and spring and completely dry in the summer and fall, when these plants flower. Thus, it is possible that genes from the coastal ecotype would be selectively advantageous at its inland locality.

Population 217 was from a grassland that was heavily influenced by fog, although it was at some distance from the Pacific Ocean in the East Bay hills. Unlike the other grassland populations we sampled, which flowered starting in July, this population flowered from May to June.

The other intermediate population, 154, grew in a brackish marsh in the Suisun Delta, where the Sacramento and San Joaquin rivers come together before they flow into San Francisco Bay. The plants from the Suisun Delta were described as a distinct taxon in the 1890s (*G. paludosa* Greene or *G.×paludosa*), and subsequently hypothesized on morphological grounds to be hybrids between inland plants that grew in the Central Valley grasslands and Inner Coast Ranges (*G. camporum* Greene) and the coastal plants that grew in the salt marshes surrounding San Francisco Bay (*G. stricta* DC. var. *angustifolia* (A.Gray) M.A.Lane/*G. humilis* Hook. & Arn.) [35], [39], [37]. Their tall stature, slightly succulent leaves, and phyllary shape are shared with the putative coastal parent, while they resemble the putative inland parent in their often more serrated leaf margins and herbaceous habit [35].

All of these intermediate populations were morphologically uniform (A. J. Moore, pers. obs.), and do not show the wide variation of morphology expected in recent, unstabilized hybrids. Their genetic intermediacy could be the signal of a hybrid origin or some lower level of gene exchange in the past.

### Plant Divergence along the Pacific Coast-To-Inland Gradient

A division into coastal and inland entities, as shown here for CA-FP *Grindelia*, has been found in many other plant groups along the Pacific coast of North America. Classic biosystematic studies found coastal and inland species or coastal and inland ecotypes of species with wide geographic ranges based on morphological and ecological differences (e.g., *Achillea*, *Artemisia* L., *Epilobium* L., *Horkelia* Rchb. ex Bartl., *Sisyrinchium* L., [2], and *Mimulus*, [56]). Clausen et al. [4] investigated the various ecotypes of the *Achillea millefolium* L. complex in great detail and found strong, genetically based, phenological and morphological differentiation along their west-to-east transect from the San Francisco Bay Area to the Sierra Nevada. Clausen [57] also observed morphological differentiation in *Layia gaillardioides* (Hook. & Arn.) DC. that was congruent with a geographical division between populations from the Outer Coast Ranges and Inner Coast Ranges.

Although biosystematic studies of CA-FP *Grindelia*, including common garden investigations, were not oriented around ecotype discovery [32], [33], comparable divisions are reflected in the taxonomy. For example, in Steyermark’s early monograph of the genus [35], coastal plants were called *G. arenicola* Steyerm., *G. blakei* Steyerm., *G. hirsutula* Hook. & Arn. (in part), *G. humilis*, *G. maritima* (Greene) Steyerm., *G. rubricaulis* DC., and *G. stricta*, while inland plants were classified in *G. camporum* and *G. hirsutula* (in part) [35]. The divisions between the putative taxa within the coastal and inland ecotypes were also largely ecologically-based (with the morphology following ecology).

For some plant groups, molecular data have reinforced or extended the ecological findings of past biosystematic studies, but for others they have not. While coastal and inland lineages of *Layia gaillardioides* were also apparent from DNA sequence data [58], phylogeographic study did not show a clear division along ecotypic lines in the *Achillea millefolium* complex [30]. In the latter case, it is possible that the coastal ecotypes originated multiple times (as found in *Eucalyptus globulus* [16] and *Senecio lautus* [17]) or that they are too young for complete lineage sorting to have taken place [59], [18]. In *Downingia yina* Applegate, molecular studies found three cryptic species along a coast-to-inland transect, although these were not suspected based on previous morphological work: *D. willamettensis* M. Peck occurs west of the Cascade Ranges (coastal), *D. pulcherrima* M. Peck occurs to the east (inland), and *D. yina* s.s. is localized in the Cascade Range [28].

The differentiation of *Mimulus guttatus* into coastal and inland ecotypes has been extensively studied, both molecularly and experimentally [18]. The ecotypes of *M. guttatus* differ adaptively, with the coastal plants being more salt tolerant [19] and the inland plants more drought tolerant [60]. In addition, the two ecotypes are reproductively isolated under natural conditions, due to little overlap in flowering time and to selection against immigrants from the other ecotype [18]. As in *Grindelia* [33], intrinsic postzygotic isolation between ecotypes was not found [18].

In contrast to the *M. guttatus* ecotypes, the coastal and inland ecotypes in CA-FP *Grindelia* have overlapping flowering periods, and they occasionally do occur close enough together for cross-pollination to occur, as at our site on Point Pinole. More study is needed to determine whether the lack of gene flow is due to one or multiple factor(s), such as hybrids simply not being formed (perhaps, for example, due to a stigmatic preference for pollen from the same ecotype) or to selection against hybrids at the seedling stage due to distinct habitat adaptations of the parents. In any case, the populations of the two plants at the site where they co-occur are just as morphologically uniform as they are in sites where the ecotypes occur alone (A.J. Moore, pers. obs.).

The origin of the coastal and inland groups in CA-FP *Grindelia* is unclear. They could potentially represent two separate tetraploidization events. They could also have arisen from greater gene exchange within the coastal group and within the inland group than between the groups (either due to geographic circumstances or to barriers to gene exchange between groups). In the one instance where we found two populations of different habitats occurring adjacent to each other without exchanging genes, the populations were from the two different ecotypic groups, but it is unclear if similar phenomena could be observed between populations of different habitats within the coastal or inland groups.

Given our limited sampling and the relatively large number of intermediate populations, it would be premature to draw any firm taxonomic conclusions. If all CA-FP *Grindelia*, or at least all of the tetraploids, are classified as one species, the oldest available name is *G. hirsutula*. If, instead, the inland and coastal groups are treated as distinct species, as could be warranted if further evidence of genetic isolation is found, the taxonomic situation is more complicated, in part because plants that have been classified as *G. hirsutula* are found in both groups, and the type specimen of *G. hirsutula* does not correspond exactly with any of the plants we have sampled thus far. If the type of *G. hirsutula* is found to belong to the coastal group, then the oldest available name for the inland group would likely be *G. camporum*. Instead, if the type of *G. hirsutula* is found to belong to the inland group, the oldest available name for the coastal group would likely be *G. stricta*.

Early differentiation of lineages in evolutionary hotspots, such as the CA-FP, poses serious challenges for resolving and classifying biodiversity. *Grindelia* is not alone in posing such difficulties, especially among young perennial lineages that retain interfertility across populations and that span ecological gradients. Such groups represent an important frontier for the application of genomic and other methods in order to understand, communicate, and protect some of the most interesting examples of recent evolutionary change.

## Supporting Information

Table S1 **Pairwise matrix of ρ distances between populations.**
(CSV)
